# Alterations in Cortical Thickness and White Matter Integrity in Mild-to-Moderate Communicating Hydrocephalic School-Aged Children Measured by Whole-Brain Cortical Thickness Mapping and DTI

**DOI:** 10.1155/2017/5167973

**Published:** 2017-01-16

**Authors:** Siyu Zhang, Xinjian Ye, Guanghui Bai, Yuchuan Fu, Chuanwan Mao, Aiqin Wu, Xiaozheng Liu, Zhihan Yan

**Affiliations:** ^1^China-USA Neuroimaging Research Institute, Radiology Department of The Second Affiliated Hospital and Yuying Children's Hospital, Wenzhou Medical University, Wenzhou, Zhejiang, China; ^2^Radiology Department, Meizhou People's Hospital, Meizhou, Guangdong, China

## Abstract

Follow-up observation is required for mild-to-moderate hydrocephalic patients because of the potential damage to brain. However, effects of mild-to-moderate hydrocephalus on gray and white matter remain unclear in vivo. Using structural MRI and diffusion tensor imaging (DTI), current study compared the cortical thickness and white matter integrity between children with mild-to-moderate communicating hydrocephalus and healthy controls. The relationships between cortical changes and intelligence quota were also examined in patients. We found that cortical thickness in the left middle temporal and left rostral middle frontal gyrus was significantly lower in the hydrocephalus group compared with that of controls. Fractional anisotropy in the right corpus callosum body was significantly lower in the hydrocephalus group compared with that of controls. In addition, there was no association of cortical thinning or white matter fractional anisotropy with intelligence quota in either group. Thus, our findings provide clues to that mild-to-moderate hydrocephalus could lead to structural brain deficits especially in the middle temporal and middle frontal gyrus prior to the behavior changes.

## 1. Introduction

Hydrocephalus is a pathologic condition in which excessive cerebrospinal fluid (CSF) accumulates in the ventricular system because of obstruction along CSF pathways or an imbalance between CSF production and reabsorption [1, 2]. The enlarged ventricles and associated increase in intracranial pressure can cause damage to various brain regions, including the gray matter (GM) and white matter (WM). Surgical treatment is presently the main therapeutic option for severe hydrocephalus. However, follow-up observation is required for mild-to-moderate hydrocephalic patients because of risk of operation [3, 4].

Although conventional MRI techniques, including T1 weighted image (T1WI) or T2 weighted image (T2WI), can detect major structural abnormalities, they cannot detect more subtle GM and WM abnormalities, such as cortical thickness and ultrastructural changes, in hydrocephalic patients during follow-up observation. By contrast, 3-dimensional fast spoiled gradient-recalled sequence can provide high-resolution T1WI data that allowed us to observe and measure cortical thickness. DTI provides quantitative information on anisotropic diffusion properties in WM and has been used to investigate WM damage and recovery in various neurologic and pathologic disorders [5–8]. Changes of cortical thickness were reported in a variety of diseases [9–11]. Severe hydrocephalus also leads to compression of the cerebral cortex, with reduction of overall brain mass and cortical thickness, particularly in the parietal and occipital regions [12–14]. In animal studies, an increasing degree of ventriculomegaly is associated with more marked thinning of the cerebral cortex [15]. However, to our knowledge, there are very few studies examining both GM and WM abnormalities in school-aged children with mild-to-moderate hydrocephalus, which is important because mild-to-moderate hydrocephalus may have a negative impact on children and reduce their quality of life in the future.

In the present study, we performed MRI studies on school-aged children with mild-to-moderate communicating hydrocephalus to identify regional changes of cortical thickness and WM fractional anisotropy (FA) and assessed the correlation between cortical changes and intelligence quota (IQ).

## 2. Materials and Methods

### 2.1. Subjects

This study included 15 treatment-naive patients with communicating hydrocephalic (mean age = 9.76 ± 1.80 years; duration of illness = 1.77 ± 0.68 years; sex ratio = 6 female/9 male). All hydrocephalus subjects were recruited from patients who visited The Second Affiliated Hospital of Wenzhou Medical University from December 2013 to June 2015. The inclusion criteria were (1) age between 6 and14 years, (2) IQ within the normal range, (3) no evidence of obstruction in CSF flow and no GRASS MRI finding of other diseases, (4) Evan's index between 0.33 and 0.50, (5) duration of illness more than 1 year, (6) no history of other intracranial or other related diseases (e.g., meningitis, head trauma, epilepsy, nephritic, congenital heart disease, and diabetes mellitus), (7) right-handed, and (8) no claustrophobia [3, 4]. The control group consisted of 20 healthy comparison subjects with age (9.50 ± 1.96 years), gender (8 female/12 male), IQ, family condition, and education level matched with patient group. Healthy control subjects were recruited through poster advertisements. Demographic and IQ data are summarized in Table 1. Informed consent was obtained from all subjects and their guardian for study participation.

### 2.2. Intelligence Assessments

The Wechsler Intelligence Scale for Children-Chinese Revised, which demonstrates high reliability and validity, was used to assess general intellectual ability of all subjects and the linguistic IQ and performance IQ, and senior medical staff that majored on intelligence assessment calculated full scale IQ.

### 2.3. Imaging Data Acquisition

MRI data were obtained on a 3.0 Tesla system (GE Signa HDxt) with an 8-channel phase array head coil. High-resolution T1WI was acquired with a volumetric 3-dimensional fast spoiled gradient-recalled sequence (slice thickness = 1.0 mm, slice gap = 0.5 mm, TR = 9 ms, TE = 4 ms, FOV = 240 × 240 mm^2^, matrix = 256 × 256, and in-plane resolution = 0.94 × 0.94 mm^2^). DTI images were acquired with a diffusion-weighted spin-echo sequence (slice thickness = 4.0 mm, TR = 8000 ms, TE = 90 ms, FOV = 240 × 240 mm^2^, matrix = 128 × 128, in-plane resolution = 1.88 × 1.88 mm^2^, and flip angle = 90°; diffusion weighting was applied with a* b* value = 1000 s/mm^2^ along 36 independent nonlinear orientations, and 1 additional image with no diffusion weighting was acquired).

### 2.4. Imaging Processing

High-resolution T1WI analysis was performed using the FreeSurfer package (http://surfer.nmr.mgh.harvard.edu/). Cortical thickness was measured as the difference between the position of equivalent vertices in the pial and GM/WM surfaces, by utilizing both intensity and continuity information from the entire 3-dimensional MR volume in the segmentation and deformation procedures. The reliability of the method was previously validated against histological analysis on postmortem brains and manual measurements, and the test-retest reliability is high [16–19]. Briefly, the main steps included motion correction using FSL FLIRT and automated registration to Talairach space, segmentation of the subcortical GM and WM structures, intensity normalization and removal of nonbrain voxels, and tessellation of GM and WM boundaries and automated topology correction and surface deformation to optimally place the GM/WM and GM/CSF borders defined at the location with the greatest shift in signal intensity [16, 20–24]. Following registration of all subjects' cortical reconstructions to a common average surface, the surface maps were interpolated and analyzed. This procedure is capable of detecting submillimeter differences between groups [16].

DTI image processing was performed using the FSL package (http://fsl.fmrib.ox.ac.uk/fsl/fslwiki/). Data were inspected for movement artifacts using FSL-MCFLIRT (<1° rotation and <1 mm translation) and then corrected for eddy current-induced distortions. Brain extraction and calculation of diffusion parameter maps were performed using FSL. FA maps for each participant were registered into a standard brain template (FMRIB58_FA, part of the FSL suite) using the nonlinear spatial transformation tool FNIRT. A mean FA image was then compiled by averaging aligned FA maps from each participant. To generate a mean FA skeleton representing the centers of all tracts common to the group, the map threshold was then set for voxels showing FA values ≥ 0.2. Aligned FA maps for each participant were projected onto the standard skeletonized FA image (FMRIB58_FA-skeleton, packaged in FSL) by searching the area around the skeleton in the direction perpendicular to each tract, finding the highest local FA value, and assigning this value to the skeleton [25].

### 2.5. Statistical Analysis

In the graphical interface of FreeSurfer (QDEC [Query, Design, Estimate, Contrast]), structural parameters were smoothed with full width at half maximum at 10 mm, mesh surface-base, and concomitant variable of age. Cortical thickness was compared between the two groups, corrected using the Monte Carlo simulation (corrected threshold = 1.3, *P* < 0.05), to find regions with significant group differences.

Using tract based spatial statistics tools, voxelwise spatial statistical analysis comparing the hydrocephalus and control groups was performed using the “randomize” program within FSL, which involves permutation testing [26]. The mean FA skeleton was used as a mask (threshold at a mean FA value of 0.2), and the number of permutations was set to 5000. Thresholding was performed using threshold-free cluster enhancement, a new method for finding significant clusters in MRI data without having to define them as binary units [27]. Clusters were assessed for multiple comparisons using the familywise error rate (*P* < 0.05).

To evaluate the relationship between cortical changes and IQ, we extracted the mean value of cortical thickness of regions with significant intergroup differences. A 2-sample* t*-test was performed to compare age and IQ of the hydrocephalus and control groups, while Chi-square test was performed to compare gender ratio (SPSS v19.0 software). Bivariate correlations were performed between cortical thickness, FA changes, and IQ for each group.

## 3. Results

### 3.1. Group Differences in Demographics

There were no significant differences in age (*P* = 0.80) or gender composition (*P* = 0.73) between the hydrocephalus and control groups (Table 1).

### 3.2. Group Differences in Cortical Thickness

**Figure 1:**
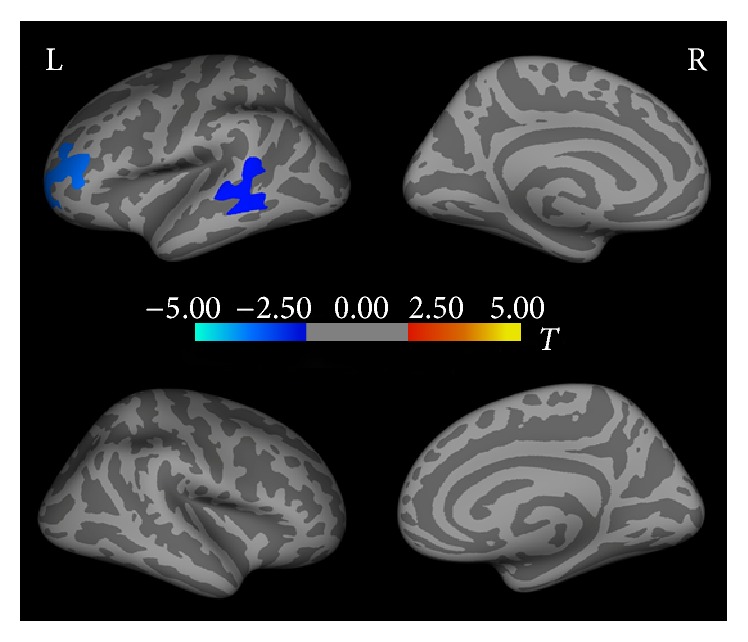
Differences in cortical thickness between hydrocephalus group and control group. Significance was determined by Monte Carlo simulation. Less cortical thickness in hydrocephalus group than in control group is indicated by blue/cool color. *P* < 0.05. L: left hemisphere. R: right hemisphere.

Compared with the control group, hydrocephalus patients had a lower cortical thickness in the left middle temporal gyrus (area = 1122.09 mm^2^; Talairach coordinates = −61.5, −40.8, −3.2; *P* < 0.05; and mean thickness = 2.37 ± 0.22 [hydrocephalus group] and 2.44 ± 0.25 [control group]) and in the left rostral middle frontal gyrus (area = 1402.96 mm^2^; Talairach coordinates = −34.6, 48.2, 6.3; *P* < 0.05; and mean thickness = 2.88 ± 0.26 [hydrocephalus group] and 2.92 ± 0.18 [control group]). There were no brain regions with increased cortical thickness in hydrocephalus patients (Figure 1, Table 2).

### 3.3. Group Differences in FA

**Figure 2:**
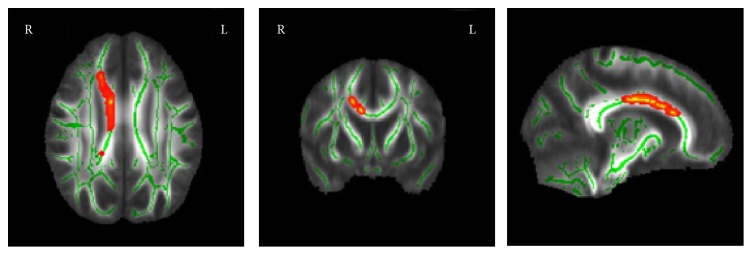
Differences in FA values between hydrocephalus group and control group. Significance was determined by* t*-test. Less FA values in hydrocephalus group than in control group are indicated by red/warm color. *P* < 0.05. L: left hemisphere. R: right hemisphere.

Compared with the control group, hydrocephalus patients had a lower FA in the right corpus callosum body (region size = 836 voxels; MNI coordinates = 11, −11, 31; *P* < 0.05; and mean FA = 0.51 ± 0.07 [hydrocephalus group] and 0.63 ± 0.09 [control group]). There were no brain regions with increased FA in hydrocephalus patients (Figure 2, Table 3).

### 3.4. Cortical and FA Changes with IQ

In the control group, there were a trend towards a decrease in cortical thickness in the left middle temporal gyrus with increasing linguistic IQ and full scale IQ and a trend towards an increase in cortical thickness in other regions with increasing linguistic IQ, performance IQ, and full scale IQ. In both the control and the hydrocephalus groups, there were a trend towards an increase in cortical thickness in the left rostral middle frontal gyrus with increasing linguistic IQ, performance IQ, and full scale IQ and a trend towards a decrease in FA in the right corpus callosum body with increasing linguistic IQ. Further, in the hydrocephalus group, there was a trend towards a decrease in full scale IQ. By contrast, there was no association of cortical thinning or decreased FA with IQ in either group (Tables 4–6).

## 4. Discussion

Unlike the widespread cortical deficits of the whole brain in patients with severe hydrocephalus, school-aged children with mild-to-moderate communicating hydrocephalus showed significant cortical thinning in the left middle temporal gyrus and left rostral middle frontal gyrus and decreased FA in the right corpus callosum body, though the two groups showed no difference of IQ. Furthermore, there was no correlation between the mean cortical thickness nor the FA and IQ in either group. These findings suggest that the gray matter of left temporal and frontal lobe and white matter of corpus callosum are the most vulnerable regions in children with mild-to-moderate communicating hydrocephalus, which may happen before the behavior changes.

Widespread cortical thinning was previously reported in animal models and children with severe hydrocephalus [13, 15, 28, 29]. This is consistent with the pathology studies, which revealed moderate-to-severe neuronal swelling of the whole brain in hydrocephalus patients, with marked enlargement of the extracellular space in the adjacent neuropil, synaptic plasticity and degeneration, damage to myelinated axons, and myelination delay. Astrocytes also display evidence of edema and phagocytic activity [29, 30]. These pathological changes in the cerebral cortex of human hydrocephalus patients are considered to result from an initial mechanical injury because of the high CSF pressure, followed by secondary changes associated with increased interstitial edema, ischemia, and oxidative stress [31]. However, the evidences for the gay and matter changes in children with mild-to-moderate communicating hydrocephalus are rare. Our findings in vivo showed that the regional decreased gray matter does happen in the left temporal and frontal lobe.

The decreased FA in the right corpus callosum body of infants and children with severe hydrocephalus was also reported in previous studies [32–37]. DTI studies in acute hydrocephalus patients revealed increased FA in the WM areas lateral to the ventricles, which recovered after surgery, suggesting white matter compression as the possible cause of this observed change, and decreased FA in the corpus callosum, with no changes after surgery, suggesting that the corpus callosum may be easier to undergo neuronal degeneration [32]. In addition, infants with hydrocephalus showed significantly lower FA in the corpus callosum, but not in the internal capsule [33]. Pathological studies on the corpus callosum in acute and chronic hydrocephalus patients suggest a potential causative role of neuronal degeneration during hydrocephalus [36, 37]. Current studies provided further evidences in vivo that the white matter deficits were prominent in the right corpus callosum body. Compared to the previous studies in patients with severe hydrocephalus, our research focused on mild-to-moderate communicating hydrocephalic children who had shorter duration and lower-level hydrocephalus than those of previous studies [13, 15, 28, 29, 32–37]. These findings suggest that the corpus callosum is the most vulnerable white matter tract under hydrocephalus, which happens early in the course of the illness.

We acknowledge several potential limitations of our study. First, our results were derived from a limited number of participants, which may have had an adverse effect on the power of statistical analysis. Second, a confounding factor is the relationship between age of onset and disease duration, which is difficult to resolve in a cross-sectional design. Thus, further longitudinal studies with more patients are required to confirm the changes in cortical thickness and WM ultrastructure in children with mild-to-moderate communicating hydrocephalus. A third limitation is that the Wechsler Intelligence Scale for Children-Chinese Revised is a basic and very ordinary intelligence scale for children, which may be the reason why no association of cortical thinning and decreased FA with IQ was found. Therefore, more detailed intelligence scales may be required to detect specific differences. In addition, we use Evan's index instead of cerebrospinal fluid pressure as crucial indicator for noninvasiveness. However, the accuracy of Evan's index is still not very ideal and more reference indexes will be helpful for noninvasive indicators [3]. Meanwhile, our study lacked functional MRI information and behavior assessments, which needs to be studied further.

## 5. Conclusions

By studying the school-aged children with mild-to-moderate communicating hydrocephalus, our findings provide evidences that the gray matter of left temporal and frontal lobe and white matter of corpus callosum are the most vulnerable regions in mild-to-moderate hydrocephalus, which happens even before the behavior changes. Thus, structural analysis by MRI can be used to monitor long-term outcomes and follow-up observation in mild-to-moderate hydrocephalic patients.

## Figures and Tables

**Table 1:** Demographics and IQ of two groups.

	Hydrocephalus group (*N* = 15)	Control group (*N* = 20)	*P* value
Gender			0.73
Male	9	12	
Female	6	8	
Age (years)	9.67 ± 1.80	9.50 ± 1.96	0.80
Duration of illness (years)	1.77 ± 0.68	—	—
Evan's index	0.35 ± 0.02	0.26 ± 0.01	<0.01
Linguistic IQ	91.47 ± 19.37	87.50 ± 8.68	0.47
Performance IQ	96.13 ± 13.93	92.60 ± 12.38	0.43
Full scale IQ	93.00 ± 17.33	88.90 ± 9.59	0.42

The data were presented as mean ± SD.

**Table 2:** Changes in cortical thickness in hydrocephalus patients.

	Region size (mm^2^)	Cortical thickness (mm)		Talairach coordinate		
		Hydrocephalus group	Control group	Tal*X*	Tal*Y*	Tal*Z*
Left rostral middle frontal	1402.96	2.88 ± 0.26	2.92 ± 0.18	−34.6	48.2	6.3
Left middle temporal	1122.09	2.37 ± 0.22	2.44 ± 0.25	−61.5	−40.8	−3.2

The data were presented as mean ± SD.

**Table 3:** Changes in FA values in hydrocephalus patients.

	Region size (voxels)	FA		MNI coordinate		
		Hydrocephalus group	Control group	*X*	*Y*	*Z*
Right corpus Callosum body	836	0.51 ± 0.07	0.63 ± 0.09	11	−11	31

The data were presented as mean ± SD. FA: fractional anisotropy. MNI: Montreal Neurological Institute.

**Table 4:** Relationship between mean cortical thickness of the left middle temporal and IQ.

	Hydrocephalus group		Control group	
	*r* ^a^	*p*	*r*	*p*
Linguistic IQ	0.368	0.177	−0.235	0.319
Performance IQ	0.051	0.858	0.046	0.849
Full scale IQ	0.255	0.359	−0.098	0.680

^a^+: increase. −: decrease.

**Table 5:** Relationship between mean cortical thickness of the left rostral middle frontal and IQ.

	Hydrocephalus group		Control group	
	*r* ^a^	*p*	*r*	*p*
Linguistic IQ	0.360	0.188	0.030	0.900
Performance IQ	0.268	0.334	0.256	0.276
Full scale IQ	0.330	0.230	0.160	0.501

^a^+: increase. −: decrease.

**Table 6:** Relationship between mean FA of the right corpus callosum body and IQ.

	Hydrocephalus group		Control group	
	*r* ^a^	*p*	*r*	*p*
Linguistic IQ	−0.230	0.409	−0.049	0.838
Performance IQ	0.054	0.847	0.235	0.318
Full scale IQ	−0.137	0.627	0.124	0.603

^a^+: increase. −: decrease.

## Abbreviations

CSF: Cerebrospinal fluid
GM: Gray matter
WM: White matter
MRI: Magnetic resonance imaging
T1WI: T1 weighted image
T2WI: T2 weighted image
3-DFSPGR: 3-Dimensional fast spoiled gradient-recalled
DTI: Diffusion tensor imaging
FA: Fractional anisotropy
IQ: Intelligence quota
TR: Repetition time
TE: Echo time
FOV: Field of view
MNI: Montreal Neurological Institute
SD: Standard deviations.
